# Immune cells: plastic players along colorectal cancer progression

**DOI:** 10.1111/jcmm.12117

**Published:** 2013-09-24

**Authors:** Giuseppe Di Caro, Federica Marchesi, Luigi Laghi, Fabio Grizzi

**Affiliations:** a Laboratory of Molecular Gastroenterology, Humanitas Clinical and Research CenterRozzano, Milan, Italy; b Department of Immunology and Inflammation, Humanitas Clinical and Research CenterRozzano, Milan, Italy; c Department of Medical Biotechnology and Translational Medicine, University of MilanMilan, Italy; d Department of Gastroenterology, Humanitas Clinical and Research CenterRozzano, Milan, Italy

**Keywords:** Colorectal cancer, inflammation, immunity, prognosis, adjuvant chemotherapy

## Abstract

Inflammatory cells are involved in tumour initiation and progression. In parallel, the adaptive immune response plays a key role in fighting tumour growth and dissemination. The double-edged role of the immune system in solid tumours is well represented in colorectal cancer (CRC). The development and progression of CRC are affected by the interactions between the tumour and the host's response, occurring in a milieu named tumour microenvironment. The role of immune cells in human CRC is being unravelled and there is a strong interest in understanding their dynamics as to tumour promotion, immunosurveillance and immunoevasion. A better definition of immune infiltration would be important not only with respect to the ‘natural history’ of CRC, but in a clinically relevant perspective in the 21st century, with respect to its post-surgical management, including chemotherapy responsiveness. While it is becoming established that the amount of tumour-infiltrating lymphocytes influences the post-surgical progression of early-stage CRC, the relevance of this immune parameter as to chemotherapy responsiveness remains to be clarified. Despite recent experimental work supporting the notion that infiltrating immune cells may influence chemotherapy-mediated tumour cell death, tumour-infiltrating cells are not employed to identify patients who are more likely to benefit from adjuvant treatment. This review focuses on studies addressing the role of innate and adaptive immune cells along the occurrence and the progression of potentially curable CRC.

- Colorectal cancer, epidemiology and staging
- The immune system in cancer
  – Cancer-related inflammation
  – Cancer-related inflammation in colorectal cancer: molecular mechanisms
  – Anti-tumour activities of the immune system in human colorectal cancer
- Immune cells and response to chemotherapy in colorectal cancer
- Concluding statements

## Colorectal cancer: epidemiology and staging

In 2008, IARC ranked colorectal cancer (CRC) second for cancer prevalence and third for mortality in men and third for frequency and second for mortality in women in developed countries. Advances in population screening strategies make possible an early detection of precancerous lesions. Accordingly, data from the Surveillance, Epidemiology, and End Results (SEER) program of the NCI (National Cancer Institute of the United States) on a population screened from 1998 to 2008 revealed a decrease in CRC incidence (−2.6%, −2.0%; for men and women respectively) and a decrease in CRC-related mortality (−2.3%, −2.1%) [1]. However, regardless of improved screening, surgical technique and adjuvant CHT, nearly a third of CRC patients experience disease recurrence. About 20–25% of patients with CRC have metastases at diagnosis, and ≍20–25% of patients will develop metachronous metastases after surgery, resulting in relatively high overall mortality rates [2]. In synthesis, CRC death parallels the incidence of metastatic disease, and in 2008, CRC was still the cause of 9% of overall cancer-related deaths in US. Surgery still represents the backbone of CRC treatment, retaining the highest effect on survival. Following curative radical surgery in patients without lymph node or distant metastasis at diagnosis, occult micro-metastases not detectable with the current diagnostic tools are thought to be the source of disease recurrence [3]. Cancer is not a single-disease entity, and the anatomical classification based on the tissue or organ where it arises was the first advance from considering all cancers as a uniform disease [4–7]. Tumour-node-metastasis (TNM) classification and staging system is conceived to allow clinical stratification of patients according to the estimated survival, to predict prognoses, to select the most effective treatments and to allocate patients to chemotherapy [6]. To date, the most relevant prognostic factor for CRC survival and recurrence is the presence of tumour cells in regional lymph nodes at histopathological examination, which identifies stage III patients [3]. However, histopathological staging lacks accuracy, as it is based on a deterministic prediction, and for this reason, an important percentage of patients at greatest risk of disease recurrence are still undetected. Accordingly, about 30% of stage II CRC patients will experience disease recurrence, while stage III patients after radical surgery, which is thought to eradicate also cancer metastasized to regional lymph nodes, exhibit recurrence rates of up to 70% [3]. Differences in CRC recurrence among studies are likely an effect of down-staging of CRC with stage III or IV respectively, because of misidentification of cancer cells, an event named ‘Will Rogers’ effect [8]. Inaccuracy in identifying CRC patients at risk reflects at least in part methodological weakness in diagnostic procedures [3, 9, 10]. Imaging techniques for pathological diagnosis still rely on the detection of macroscopic tumour lesions, overlooking micrometastasis that results later in metachronous metastasis, although few experimental data are available to date on this issue. Conceptually, the limited accuracy of traditional TNM staging system in predicting outcome lies in the estimation of tumour progression as a merely linear process, without taking into consideration the complexity of cancer evolution as a balance of intrinsic and extrinsic determinants. To move to a more personalized cancer medicine, there is the need for molecular classifications that are able to identify patients with specific molecular patterns, progression rates and responsiveness to chemotherapy, with the aim of optimizing drug treatments. The opposing effects of inflammatory and adaptive components of the immune system in solid tumours are well represented in CRC, a tumour strongly sustained by inflammatory mechanisms [11, 12] and greatly infiltrated by adaptive cells with anti-tumour properties [13–16]. In the next chapters, we aim to discuss the differing roles of immune cells along the progression of curable CRC.

## The immune system in cancer

### Cancer-related inflammation

Rudolf Virchow, 150 years ago, was the first who described the presence of infiltrating leucocytes in tumours, theorizing that cancer arises at chronic inflammation sites [17]. In support of this view, chronic infections have been later described as linked to about 15–20% of tumours [18, 19]. Hepatitis B and C viruses, Helicobacter pylori, and papilloma virus are established risk factors for increased prevalence of hepatocellular, gastric and cervical cancer respectively. Moreover, smoking and obesity have been associated with an increase of 20% and 30% of risk of cancer respectively [20], and together with drinking alcohol can in turn cause an inflammatory status in the lungs and liver, which is thought to support the onset of cancer [21–23]. According to such data, inflammation has been recognized as an important factor in enhancing cancer occurrence and it has been recently integrated as a new ‘hallmark of cancer’ [24]. Solid cancers are structures resembling the organ from which they arise and their occurrence and progression are modified by the behaviour of immune cells recruited in the tumour microenvironment [17]. Accordingly, the crosstalk between tumour cells and their milieu is crucial along cancer progression [12]. Inflammation can affect the tumourigenesis by modulating a variety of processes, including cellular proliferation, rate of mutagenesis, inhibition of apoptosis and angiogenesis. Soluble mediators, including inflammatory cytokines and chemokines, play a crucial role in such processes [12]. Overall, extensive experimental, epidemiological and clinical data suggest that chronic inflammation is causally linked to cancer occurrence [25], with colon cancer being one of the paradigms of the connection between inflammation and cancer.

### Cancer-related inflammation in colorectal cancer

In the gut, chronic inflammation has been found to be a risk factor for CRC occurrence. Crohn's disease and ulcerative colitis, two inflammatory bowel diseases (IBD), are both associated with an increased risk to develop colitis-associated CRC (CAC). Ulcerative colitis patients have an increased risk of developing CRC depending upon the duration of active disease (2%, 8% and 18% after 10, 20, 30 years of active disease respectively) [26], and the relative risk of developing CRC did not differ between patients with Crohn's colitis and with ulcerative colitis of similar severity [27]. The pathogenesis of IBD seems to be related to an excessive stimulation of the immune system directed towards antigens of the gut microbiota leading to chronic inflammation [28]. General consensus exists on the view that chronic inflammation of the colon increases the risk of developing CRC. However, it is worth considering that CAC is expected to account for less than 2% of all CRC [29]. Excluding IBDs, the role of inflammation in sporadic CRC is rather vague from a clinical and an experimental point of view [30]. The evidence for cytokine-regulated tumour promotion comes from studies in a mouse model of CAC [31]. However, according to Terzic *et al.,* similar mechanisms might be involved in the connection between inflammation and sporadic CRC [12]. Among the most important inflammatory mediators, tumour necrosis factor (TNF)-α and interleukin (IL)-6 are drivers of cancer-associated inflammation, by activating nuclear factors NFKB and STAT3 and inducing PTGS2 (prostaglandin G/H synthase and cyclooxygenase, also known as COX-2) [12, 23, 32]. Colorectal cancer cell-lines retain constitutive expression of NFKB and STAT3 transcription factors, which are thought to be essential components of inflammatory pathways [33, 34]. However, as no activating mutations in NFKB or STAT3 have been detected to date in colorectal or colitis-associated tumours, it is likely that signalling pathways of these components are activated upstream of such transcription factors, or alternatively that they are activated in a paracrine or autocrine fashion [12]. These signalling pathways have the ability to induce an inflammatory network in the tumour microenvironment, which might result in an influential role in tumour progression [23, 32]. In clinical studies, the most convincing association between inflammation and risk of developing sporadic CRC comes from an old drug. Many robust epidemiological studies, both observational and randomized-controlled ones, have reported that the consistent intake of non-steroidal anti-inflammatory drugs (NSAIDs) like aspirin is associated with a lower probability of developing gastrointestinal cancer [35–38]. This effect is supposed to be mediated through abrogation of chronic inflammation. Non-steroidal anti-inflammatory drugs specifically target cyclooxygenases PTGS1 (prostaglandin G/H synthase and cyclooxygenase, also known as COX-1) and PTGS2, which are essential players in the induction of inflammatory pathways [39]. Accordingly, many studies have shown that PTGS2 plays a significant role in cancer development by promoting inflammation and cell proliferation [40]. In this regard, an epidemiological study from Chan, with a cohort of more than 30 thousands women, showed that those with very high plasma levels of TNFRSF1B protein had a higher risk of CRC, and the chemo-protective effect of aspirin was retained only among women with high TNFRSF1B protein levels [30]. This is an associative evidence supporting the hypothesis that aspirin reduces risk of colorectal neoplasia through inhibition of inflammatory pathways only in selected subgroups of patients. On the other hand, it is important to underline that the effect of aspirin on the survival after CRC diagnosis is still debated, raising doubts on aspirin use as an agent for adjuvant therapy in CRC. Data from a recent paper from Liao *et al*. show that regular aspirin use on patients after CRC diagnosis had an impact on postsurgical survival only in the subgroup of patients with PIK3CA (phosphatidylinositol-4, 5-bisphosphonate 3-kinase catalytic subunit alpha gene) mutations in the tumour [41]. Mutations in PIK3CA are found in only about 15–20% of CRC [41, 42]. Therefore, NSAIDs' protective effect in the progression of CRC seems to be retained only among subclasses of patients with peculiar molecular features, supporting a ‘tailored’ role of chronic inflammation in sporadic CRC pathogenesis, from onset up to its progression. Overall, considering the heterogeneity of CRC behaviour among patients and the complex interactions between tumour and immune cells in cancer microenvironment, it is challenging to define the role of immune cell types, cytokines or growth factors in promoting or containing cancer. Thus, caution is required when assessing the contribution of mediators of inflammation to cancer biology, as they might reveal variable roles along tumour progression, even within the same organ.

### Anti-tumour activities of the immune system in colorectal cancer

**Fig. 1:**
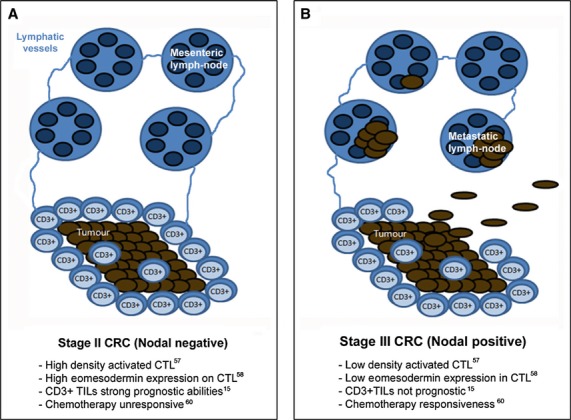
Immunological and histological features and therapeutic efficacy of Stage II *versus* Stage III colorectal cancer (CRCs). (**A**) No cancer cells infiltration is detectable in stage II CRC draining mesenteric lymph nodes. (**B**) Stage III CRCs have at least one diagnosed metastatic mesenteric lymph node derived from the primary tumour.

Besides chronic inflammation, which is thought to be critically involved in tumour occurrence, experimental and clinical evidence has revealed a protective role for immune cells along cancer progression. It was the possibility of developing new genetic models of immunodeficiency that in the early 1990s readdressed the understanding of the roles of immunity in cancer. The idea that the immune system controls tumour outgrowth, namely cancer immunosurveillance, resides in the property of cancer cells to express antigens that are not expressed by the normal tissue from which they arise. Seminal experimental models indirectly demonstrated the presence of tumour antigens, which were named ‘transplantation rejection antigens’ [43]. This evidence led to the assumption that the immune system has a dual role on cancer evolution, eliminating or promoting it. In 2002, Schreiber *et al*. postulated the cancer immunoediting theory proposing three phases of immunosurveillance: elimination, equilibrium and escape [44]. In the elimination phase, adaptive and innate immune systems interact to detect tumour antigens and to eliminate it. In the second phase, the immune system and tumour cells get into an equilibrium, wherein tumour immunosurveillance restrains, but does not completely eliminate a population of tumour cells in constant clonal evolution, thus shaping tumour cells immunogenicity alike in a Darwinian selection process. More aggressive tumour clones are selected through waves of adaptations necessary to evade innate and adaptive immune defence [45], thus contributing to tumour progression [46]. Significant variations occurring between murine and human immune repertoire, the lack of experimental models properly recapitulating CRC disease progression and the great range of immune infiltration extent among human CRC with the same histological features paved the way for several ‘natural experiments’ based on epidemiological evidence. Human immunodeficiency (*i.e*., AIDS) patients have a higher risk of colon, pancreas, lung, kidney, head and neck and melanoma cancers [46], which is a clinical evidence supporting the relevance of cancer immunosurveillance. Moreover, reports exist of patients receiving organ transplantation and later developing tumours identical to those that previously had affected the donor, who had been treated and recovered [47]. A plausible explanation for this evidence is that tumour cells were present in the donor, even though not clinically detectable, and were kept in a dormant state from donor's immune system during the equilibrium phase. The transplant of such cells in an immunodepressed and naïve host gave the ability to cancer cells to grow and become clinically evident. Although the detailed nature of CRC-related antigens has yet to be determined, many clinical studies related the varying extent of immune cells to the prognosis of patients [13–15, 48–56]. However, most of these studies were designed to demonstrate the independent association of immune markers with CRC relapse and survival by their histopathological features. In contrast, a study from Koch *et al*. functionally verified on CRC human tissues that activation and cytotoxic activity of CD8^+^ TILs were tumour specific and responsive to MUC1 [57], a tumour-associated antigen (TAA) expressed by most CRC as non-self-antigen. In this study, the authors found higher percentage of activated CTL CD8^+^ TILs in CRC tissues compared with their normal counterpart, and their activation, cytotoxic activity and reactivity were correlated with the presence of functional tumour specific reactive T cells in the blood and bone marrow. Importantly, they found that the proportion of activated TILs decreased significantly along tumour stage (from stage II through stage III-IV), showing functional decrease in immunosurveillance together with CRC progression, as depicted by histopathological staging [57]. Atreya *et al*. proposed that the proportion of activated CD8^+^ TILs and their cytolytic ability are central to mediating an effective anti-tumour activity [58, 59]. Authors demonstrated that the expression of EOMES (eomesodermin), a T-box transcription factor, was inversely correlated with the presence of lymph node metastasis at diagnosis and that was crucial in controlling the production of perforin by CD8^+^ CTLs and thus enhancing their cytotoxic activity [58, 59] (Fig. 1). In line with this clinical evidence, a paper from Laghi *et al*. showed a clinical phenomenon consistent with cancer immunoescape theory along with the progression of CRC from stage II to stage III [15]. In this study, CD3^+^ densities lost their prognostic ability in CRC patients with nodal metastasis, while they were a strong prognostic factor for occurrence of metachronous metastasis in patients without lymph-nodal involvement at diagnosis. This paper provides phenomenological and clinical evidence that the progression of CRC across TNM stages parallels the need for cancer cells to undergo immune evasion (Fig. 1). Under this respect, cancer immunoediting theory fits with the clinical behaviour of sporadic CRC. Surgery usually removes macroscopically detectable CRC burden by physical excision, while adjuvant chemotherapy is administered by assuming that it will kill circulating tumour cells and micrometastasis. Such cells are not detectable by conventional diagnostic methods and are likely in a dormant state, while after a given time, they may give rise to metachronous metastases, the main cause of death in CRC. Immune system might keep micrometastasis in an equilibrium phase for many years, and the clonal evolution of tumour cells gives rise to clones retaining the ability to escape immune recognition and cause recurrence. In this setting, it is important to underline that adjuvant chemotherapy gives a survival advantage only to stage III but not to stage II CRC patients with or without poor prognostic features [60]. On the other hand, stage IV CRC patients not receiving radical surgical treatment, if any, undergo chemotherapy as palliative medication [61, 62]. Accordingly, when taking into account stage I to stage III CRCs, chemotherapy seems to have a beneficial effect only at later stage of disease, when tumour clones are likely to have spread elsewhere. Thus, it is tempting to speculate that chemotherapy seems to have an anti-tumour effect only at a stage of disease when immunoescape mechanisms are more likely to occur, selecting tumour clones not detectable by the immune system for proper elimination (Fig. 1). In this view, it is also tempting to hypothesize that chemotherapy might enhance the *de novo* expression of molecules that might restore tumour cell immunogenicity and their recognition by immune system. It is important to notice that despite clinical evidence, the biological bases of discrepancies in terms of chemotherapy benefit along CRC progression are still unknown. Moreover, to date, CRC antigenic profile has yet to be described, and the lack of experimental models correctly reproducing CRC progression might explain at least in part our lack of knowledge.

## Immune cells and prediction of responsiveness to chemotherapy in colorectal cancer

Chemotherapy might have several impacts on the immune system. Although chemotherapy may lead to clinically relevant myelosuppression [63], it might also induce immunogenic cell death, involving the *de novo* expression of immunogenic neo-antigens on tumour cells, thus boosting immune cells' anti-tumour abilities [64–68]. In CRC, many clinical studies depicted densities of infiltrating immune cells as positive prognostic factors [13–15, 48–53]. However, very few studies assessed the predictive value of immune cells on the effectiveness of chemotherapy in CRC and did not provide convincing data. The need for biomarkers predictive of response to chemotherapy would have extremely important clinical implications and although these are required with great expectations, prudence should be applied when they are proposed to the medical and scientific community. In the study from Laghi, CD3^+^ cells density did not influence stage III patient's survival treated with fluorouracil adjuvant therapy [15]. In contrast, three studies (Prall *et al*., Morris *et al*., and Halama *et al*., respectively) reported association between chemotherapy clinical response and the extent of adaptive immune cells in CRC patient's prognosis [69–72] (Table 1). The first and the second study did not report any effect modification of the predictive abilities of adaptive immune cells by properly assessing their statistical interaction with chemotherapy treatment [69, 72], while the latter lacked a control group of untreated CRC patients [70, 71]. The estimation of prognostic markers by using subgroup analyses requires careful examination and proper design. Accordingly, a differential prognostic ability of a marker in distinct subgroups of patients is not evidence that the prognostic effect of the marker differs according to the variable which identifies subgroups. To prove an effect modification of immune cells on chemotherapy treatment in predicting prognosis, it should be indicated whether an interaction between these two variables exists at multivariate analysis [73]. The study by Morris showed that in an adjusted analysis, stage III colon cancer patients with higher densities of tumour-infiltrating lymphocytes were gaining a survival advantage from adjuvant chemotherapy [69]. It should be noted that in this study the assessment of lymphocyte density was obtained by pathological assessment, and not by taking advantage of computer-assisted image analysis. This technology has the advantage of providing continuous quantification for immune cells, making data informative, detailed and statistically relevant to quantify the proper threshold values. Moreover, in the two studies from Morris and Prall respectively, it is counterintuitive that 5-FU chemotherapy was significantly associated with a better survival in both subgroups of patients with low or high lymphocytes [69, 72]. The study from Halama analysed immune infiltration and prognosis in stage IV CRC patients who received palliative chemotherapy treatment [70, 71] (Table 1). It has to be considered that the absence of a ‘control arm’ of chemotherapy untreated patients impedes the assessment of any effect modification of immune cells on chemotherapy responsiveness. This problem is critical as cancer patients within the so-called ‘natural history’ scenario of the disease are no longer seen in the 21st century, and prospective randomized-controlled clinical studies necessary to address such an issue are ethically unfeasible. In this study, immune infiltration at the border of hepatic metastases was predicting better prognosis among CRC patients who received palliative chemotherapy [70, 71]. Immune infiltrate in CRC of stage IV primary tumour at diagnosis has been reported to be scarce [48], possibly reflecting immunoescape [74] in patients with liver metastases and dismal prognosis. Surgery of metastases in the liver is unlikely to be radical, thus being a potential confounding factor in prognostic assessment of immune cells. Microsatellite Instability (MSI) is a peculiar carcinogenesis pathway of genomic instability occurring in about 10% of the overall population of CRC [75]. Clinically, MSI in CRC was shown to be a molecular negative predictor of responsiveness to chemotherapy treatment [76, 77], despite being characterized as having a much higher extent of lymphocytic infiltration in the tumour stromal [15, 50, 51, 78] together with a lower metastatic potential at diagnosis [75]. This evidence is counter-intuitive in the light of preclinical data in CRC showing that chemotherapy positively interacts with adaptive cells' anti-tumour activities [64–68]. Ogino *et al*. pointed out that, according to the intrinsic molecular heterogeneity existing also in cancers with the same histopathological features, studies assessing the prognostic impact of immune infiltration should take consideration of relevant molecular features such as PIK3CA and MSI as potential confounding factors [79]. The study from Dahlin *et al*. provided evidence that MSI prognostic advantage might be dependent on the extent of CD3^+^ immune cells [80]. The interactions of these important CRC features in the context of chemotherapy treatment and stage of disease, however, remain to be uncovered. The low prevalence of molecular biomarkers is a critical issue and, as previously suggested [79], future studies should be designed by taking advantage of large cohorts of CRC patients and standardized methodologies. Thus, despite preclinical evidence, clinical studies available to date in the literature are not enough supportive of a role for adaptive immune infiltrate on the efficacy of chemotherapy treatment in CRC. Better understanding of the molecular pathways leading to the chemo-responsiveness may generate new strategies or new cellular mediators able to enhance chemotherapy-driven anti-tumour activity.

**Table 1:** Studies addressing the predictive abilities of immune cells in CRC patient's responsiveness to chemotherapy treatment

Author (year)	Ref.	No. of patients	TNM stage	Control arm*	Effect modification	TILs markers	TILs quantification
Halama (2011)	[70]	101	IV	No	ND	CD3^+^, CD8^+^, GZB^+^ or FOXP3^+^TILs	Computer assisted
Laghi (2009)	[15]	286	II-III	Yes	No effect	CD3^+^TILs	Computer assisted
Morris (2008)	[69]	1156	III	Yes	ND	None	Semi-quantitative
Prall (2004)	[72]	152	III	Yes	ND	CD8^+^TILs	Computer assisted

* CRC Patients not receiving adjuvant chemotherapy treatment.

ND: not determined; CRC: colorectal cancer.

## Concluding statements

The evidence that CRC is a heterogeneous, multifactorial disease with different outcomes, prognosis and/or response to treatments in histologically equivalent tumours is suggestive of the complexity of cancer behaviour along its progression. Even the most refined experimental models of CRC available to date do not mimic the diversity of immune infiltration among human patients. The idea that chemotherapy treatment is effective only at advanced stages of disease is overlooked in both clinical and preclinical studies relating immune cells and CRC prognosis. Translational and clinical studies to understand whether adaptive immune cells or other cellular players control the growth of CRC micro-metastasis and their interactions in the setting of chemotherapy are warranted. Acknowledgement of the dual roles of the immune system, from the onset of CRC along its progression, will help design and develop consistent strategies in preclinical and clinical investigation that might ultimately result in a better clinical management of patients.
